# Interprofessional versus monoprofessional case-based learning in childhood cancer and the effect on healthcare professionals’ knowledge and attitudes: study protocol for a randomised trial

**DOI:** 10.1186/s12913-020-05980-2

**Published:** 2020-12-04

**Authors:** Martha Krogh Topperzer, Marianne Hoffmann, Hanne Bækgaard Larsen, Susanne Rosthøj, Jacob Nersting, Louise Ingerslev Roug, Peter Pontoppidan, Liv Andrés-Jensen, Birgitte Lausen, Kjeld Schmiegelow, Jette Led Sørensen

**Affiliations:** 1 Paediatric Oncology Research Laboratory, Department of Paediatrics and Adolescent Medicine, Rigshospitalet, University of Copenhagen, Blegdamsvej 9, 2100 Copenhagen, Denmark; 2 Department of Paediatrics and Adolescent Medicine, Rigshospitalet, University of Copenhagen, Blegdamsvej 9, 2100 Copenhagen, Denmark; 3 Section of Biostatistics, Faculty of Health Sciences, University of Copenhagen, Øster Farimagsgade 5, 1014 Copenhagen, Denmark; 4 Juliane Marie Centre, Rigshospitalet and Department of Clinical Medicine, University of Copenhagen, Copenhagen, Denmark

**Keywords:** Interprofessional education, Continuing professional education, Childhood cancer, Team collaboration

## Abstract

**Background:**

Interprofessional education in childhood cancer is a multifaceted field involving multiple healthcare professionals with general and specialised knowledge and skills. Complex treatment, care and rehabilitation require continuous professional development and maintenance of healthcare professionals’ competencies in their field of expertise. However, limited knowledge exists in comparing interprofessional and monoprofessional education. Only a few randomised studies have evaluated the effectiveness and efficiency of interprofessional education.

The objective of this single-centre, investigator-initiated cluster randomised trial is to study the effect of interprofessional versus monoprofessional case-based learning on healthcare professionals’ knowledge of gastrointestinal side effects and attitudes towards team collaboration.

**Methods:**

This study will randomise healthcare professionals to participate in either the experimental interprofessional group or the control monoprofessional group of case-based learning. The topic of the case-based intervention will be gastrointestinal side effects, one of six categories identified in a three-round Scandinavian Delphi study as relevant for interprofessional education in childhood cancer.

The primary outcome is the self-reported questionnaire Assessment of Interprofessional Team Collaboration Scale. Secondary outcomes are measured by the self-reported questionnaires Readiness for Interprofessional Learning Scale Questionnaire, Safety Attitudes Questionnaire, and knowledge will be evaluated using a multiple-choice quiz.

Participants will receive the self-reported questionnaires about 2 weeks before and 1 month after the intervention. On the day of the intervention, participants will answer a multiple-choice quiz before and after the case-based learning.

Linear mixed models will be used to compare differences between the two groups in mean scores postintervention, adjusting for preintervention scores.

**Discussion:**

This study will provide insight into the differences between interprofessional and monoprofessional case-based learning and how it affects healthcare professionals’ knowledge of gastrointestinal side effects and attitudes towards team collaboration.

**Trial registration:**

The intervention was registered at Clinical Trials.gov: NCT04204109 on December 102,019 and with the National Committee on Health Research Ethics: H-19087506 December 112,019 and the Danish Data Protection Agency: P-2019-637 October 152,019.

**Supplementary Information:**

The online version contains supplementary material available at 10.1186/s12913-020-05980-2.

## Background

Interprofessional education in childhood cancer is a multifaceted field involving multiple healthcare professionals with general and specific knowledge and skills. Complex treatment, care and rehabilitation require continuous professional development and maintenance of healthcare professionals’ competencies in their field of expertise to provide children and adolescents with cancer and their families the best possible treatment and care [1].

There has been a shift in the treatment paradigm in childhood cancer from cure towards normality and interprofessional collaboration in childhood cancer research has contributed to this [2, 3]. Four out of five children with cancer will survive [4], but half of all children with acute lymphoblastic leukaemia are still affected by at least one of 14 severe toxic, treatment-related side effects [5]. Moreover, the burden of mortality in childhood cancer survivors is high and continues in adulthood [6].

To continuously ensure and strengthen high-quality treatment and care, interprofessional education must be strategically planned based on a curriculum involving all relevant healthcare professionals and specific learning outcomes [7]. This necessitates a curriculum framework comprising problem identification, needs assessment, aims and objectives, educational strategies, implementation, assessment and evaluation, and feedback [7].

In a scoping review, we described a lack of well-structured and evaluated interprofessional education programmes [8]. Postgraduate education at the department of paediatric oncology, Rigshospitalet, University of Copenhagen is organised monoprofessionally, with no formal curriculum for ensuring delivery of safe, high-quality interprofessional healthcare to this group of children, adolescents and their families.

In a three-round Scandinavian Delphi study to establish consensus on learning objectives for an interprofessional education programme in childhood cancer [9], 30 designated experts from 12 out of 14 childhood cancer departments in Denmark, Norway and Sweden identified 168 learning objectives in six categories: 1) acute life-threatening situations, 2) gastrointestinal side effects, 3) pain, 4) palliation, 5) play and activity, and 6) prescription and administration of medicine.

The learning objectives in the second category: gastrointestinal side effects will inform the case and the multiple-choice quiz that will be applied in this trial (see supplemental material for learning objectives).

This trial will focus solely on the second category as gastrointestinal side effects are frequent and potentially severe clinical problems in childhood cancer that involve multiple healthcare professionals.

The objective of this trial is to study the effect of interprofessional versus monoprofessional case-based learning (CBL) on healthcare professionals’ knowledge of gastrointestinal side effects and attitudes towards team collaboration.

The hypothesis is that interprofessional case-based learning (ICBL) will improve healthcare professionals’ knowledge of gastrointestinal side effects and attitudes towards team collaboration.

## Methods

### Trial design

**Fig. 1:**
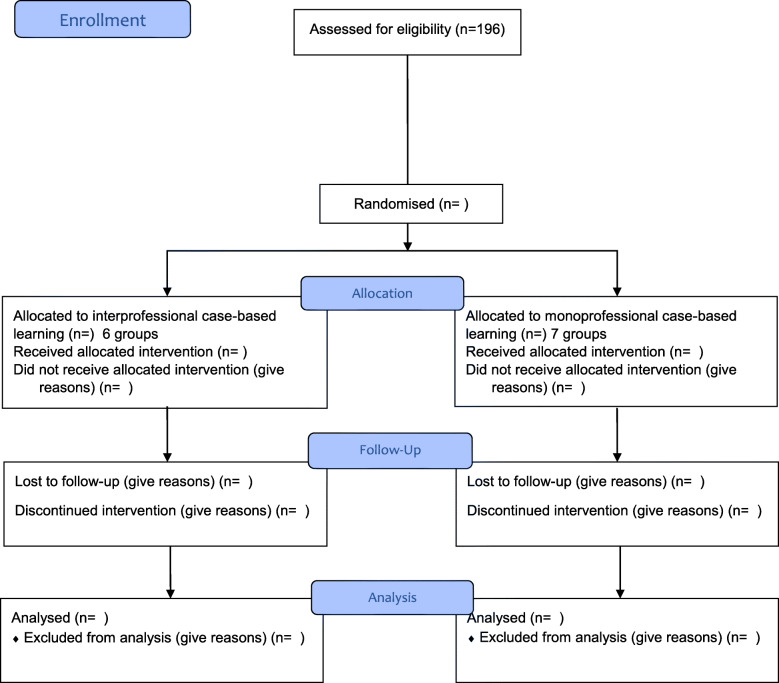
Flowchart of the progress of the phases in the randomised controlled trial

In this randomised controlled trial, the experimental group are interprofessional groups receiving CBL on children and adolescents with cancer and gastrointestinal side effects. The control groups are monoprofessional groups of either nurses or doctors. See Fig. 1 for a flowchart with an overview of the phases in the randomised controlled trial. The two groups will be taught using the same case.

### Setting and eligibility criteria

**Fig. 2:**
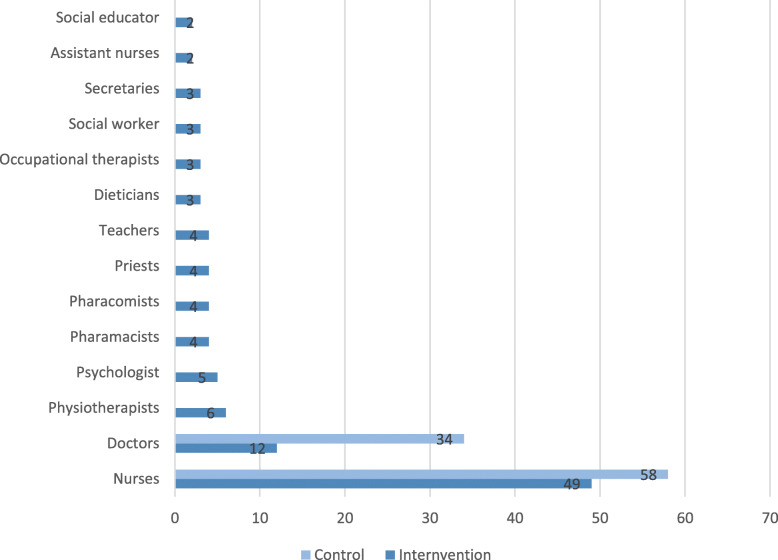
Occupational background of the participants

This randomised controlled trial will take place at Rigshospitalet, University of Copenhagen and include eligible healthcare professionals from four departments: inpatient department for children and adolescents with cancer; inpatient department for transplantation of children and adolescents with cancer; and two outpatient departments for children and adolescents with cancer (Fig. 2 provides an overview of eligible participants). Professionals employed elsewhere includes priests, pedagogues (pre-school teachers), physiotherapists, social workers, teachers, occupational therapists and dieticians.

### Exclusion criteria

Staff managers and staff involved in organising the study will be excluded from the intervention.

### Participant withdrawal

Participants will be informed by the principal investigator that they can withdraw their consent and discontinue their participation at any time. If any data has already been obtained, participants will be asked to give permission to include their data.

### Recruitment

All eligible healthcare professionals will be informed individually or in groups about the trial (for information material, please contact the corresponding author). Participants will be encouraged by the head of departments to participate in the programme.

### Details of the intervention

The intervention design is based on the CBL literature [10–12] and the research team’s didactic experience. To answer the study aims of increasing the healthcare professionals’ knowledge of gastrointestinal side effects and attitude toward team collaboration, we developed a case based on national guidelines, standard operating procedures and the identified learning objectives from the Delphi study [9]. The CBL method originating from the Harvard Business School engages participants in a structured manner towards uncovering decisions process’ and actions patterns [13]. The CBL method is ideal for healthcare professionals as discussions are part of the complex clinical decision-making process in healthcare [14, 15]. CBL sessions will not be used to teach traditionally from facilitator to healthcare professional. Rather, the didactic approach includes facilitators encouraging participants to think aloud, explain their rationales, observations, considerations and suggestions. The facilitators will guide the participants’ thought process through the structure of clinical decision-making by posing questions, eliciting opinions and stimulating a discussion, so the participants themselves explore their knowledge and attitudes [13]. For examples of learning objectives applied, see Table 1.

**Table 1:** Example of learning objectives for the CBL session

In relation to gastrointestinal toxicities and side effects, **ALL PROFESSIONALS** (teachers, pedagogues, social workers, physiotherapists, all medical doctors, nurses and other professionals affiliated with your clinic) should be able to:	
1. Identify one’s own professional limitations and ask for help	
2. Identify need for communication with the nurse and/or doctor in charge of patient	

At the core of the case method will be a real patient situation based on anonymised data containing no identifiable traits. The case will be open to interpretation as it is imperative to give as many possible alternative interpretations to what caused the problem and potential solutions (see Table 2 for exempt from the case).

**Table 2:** Exempt from case

Day 1 Monday	
Larry is 14 years ord. His has a high risk Acute Lymphatic Leukaemia (ALL) and follows the treatment protocol of ALL2gether. Today, Larry is admitted to the day hospital for his day 22 chemotherapy, vincristine as a bolus and Daunorubicin over 1 h.	
While the nurse administers the chemo to Larry’s central iv line, she asks how he is feeling. Larry tells her that his tummy hurts.	
Larry’s mom tells the nurse that she does not think Larry has had stools in 2 days. He does not want to talk about it. Larry’s mom also says that Larry’s legs hurt and that he does not have the strength to come out off bed at home. All he does is lie in the sofa and watch his Ipad. When he does come out of bed, he drags his legs behind him. This morning it took his mom3 h to get out of bed.	

A board or flip-overs will lead and synchronise the work of the group in a structured manner (see Table 3 Board illustration). The columns on the board stem from heuristic clinical problem solving (definition of problems, gathering of facts, hypothesis, hypothesis testing and feedback).

**Table 3:** Board illustration

Important facts: This we know	Problems: This we are not satisfied with	Possible explanations:	Additional information required:	Suggestions for procedures, treatment, etc.:	Study questions:
		Prognosis if nothing is done:		Anticipated effect of measures and procedures:	

The intervention consists of three and a half hours of CBL. Table 4 present a tentative programme. The intervention will be arranged at least 3 months in advance on a specific day in the healthcare professionals’ work schedule and take place during regular working hours. Participants will receive their regular salary. Participation is voluntary; however, participation is endorsed and planned in coordination with staff management.

**Table 4:** Tentative programme for intervention and control session with quiz and evaluation

Programme for case-based learning session		
Start and stop	Lecture and didactics	Quiz and evaluation
8:00–8:30	Welcome and introduction	Multiple-choice quiz
8:30–10:30 (with one break)	Case-based learning	
10:30–11:15	Recap of relevant guidelines	Multiple-choice quiz
11:15–11:30	End of lecture	Course evaluation

The research team designing the case represents the two largest professional groups in childhood cancer: doctors and nurses. The facilitators will be supervised and supported by the researchers (MKT, JLS) on how to build an atmosphere conducive to facilitating CBL in an interprofessional setting [14].

The participants in the experimental group will be randomised into six interprofessional teams. The computer-generated allocation sequence will be designed to ensure an adequate composition of health professionals resembling authentic clinical teams of 10–18 people. For example, four nurses, two doctors, one physiotherapist, one priest, one teacher, one social worker and one pharmacist.

The participants in the control group will be randomised into four monoprofessional groups comprised exclusively of nurses and three groups exclusively of doctors (See Fig. 2).

**Fig. 3:**
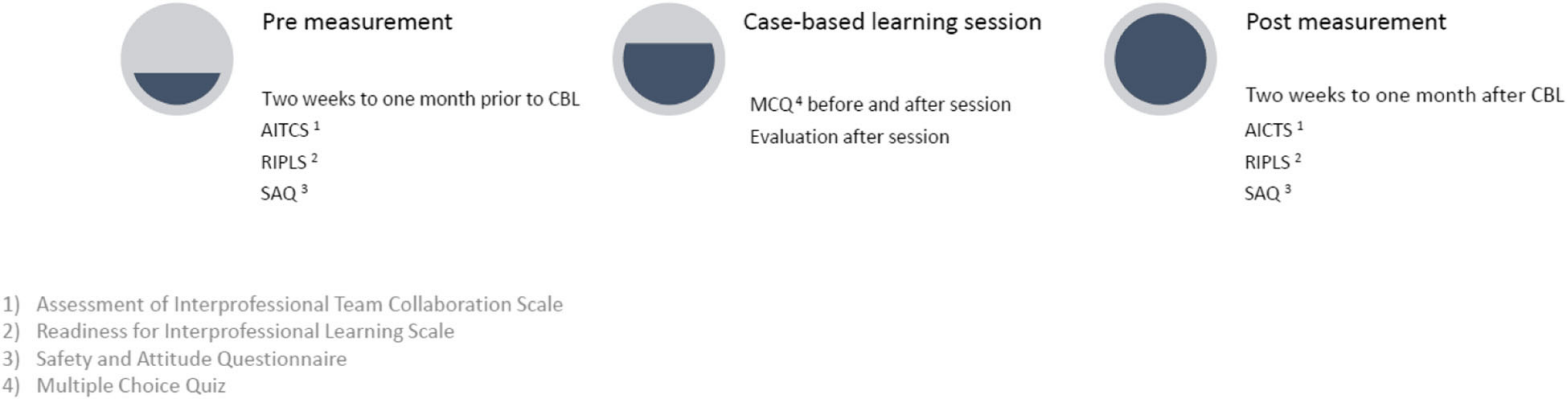
Timepoints of measurements

Two weeks to one month before the CBL session, the participants will receive an email with a link to the three questionnaires (AITCS, RIPLS and SAQ) generated in the secure web application REDCap [16]. A follow-up questionnaire will be sent to the participants 1-3 months after the intervention with a link to the same three questionnaires (see Fig. 3 for Timepoints of measurements).

An MCQ will test the participants’ knowledge before and after the CBL (see an example of MCQ item in Table 5). It will consist of one best answer format with three options will be developed for this trial based on guidelines for designing and developing questionnaires [17, 18]. The items in the multiple-choice quiz will be based on content in national guidelines. The national guidelines will be distributed to the participants before the CBL session by email as course material and will be available online [19].

**Table 5:** Example from MCQ and example from course evaluation

Lesley 11 years old is in the hospital school while waiting to the results on blood samples. With her she has her medication box from home. Suddenly Lesley has a bit of a stomach-ache and would like her pain killer that she can take when she needs it. She just does not remember what it looks like.				
Who should the teacher contact:				
1. The nurse from the outpatient clinic				
2. The doctor from the outpatient clinic				
3. Lesley’s mom				
The professional content of the course was overall (only one mark):				
Very poor	Poor	Acceptabel	Good	Very good
1	2	3	4	5

### Pilot testing

The case and the multiple-choice quiz will be pilot tested on a group of healthcare professionals that resemble the interprofessional group. The facilitators of the CBL will be two doctors and two nurses who work with education, supervision and introduction of experienced new staff from the paediatric oncology department. The multiple-choice quiz will be face- and content validated [18].

The participants will be asked whether they participated in any other childhood cancer CBL interventions between enrolment in the trial and answering the final questionnaires.

For exploratory analysis, the CBL sessions will be audio-recorded, and field notes will be taken focusing on the perceived dynamics and interactions in the room [20].

### Outcomes

For primary and secondary outcomes, we will use self-reported questionnaires, using a five-point Likert Scale. All scales are professionally translated and validated for a Danish context [21–23].

#### Primary outcome

Assessment of Interprofessional Team Collaboration Scale (AITCS) is a self-reported assessment of team collaboration for healthcare professionals [24]. We chose this instrument as it is one of the rare instruments that measure interprofessional team collaboration in qualified healthcare professionals. The Danish version is from 2011 and consists of three subscales; 1) Partnership/shared decision making (19 items), 2) Collaboration (11 items), 3) Coordination (7 items) [21, 22]. The items are distributed on a 5-point Likert scale, 5 = always, 4 = most of the time, 3 = occasionally, 2 = rarely, 1 = never). The difference will be measured on the overall scale.

#### Secondary outcomes

Readiness for Interprofessional Learning Scale (RIPLS) is a self-reported assessment of readiness of interprofessional learning [25]. We will use the Danish version of RIPLS with four subscales and 29 items distributed on a 5-point Likert scale, 5 = strongly agree, 4 = agree, 3 = neutral, 2 = disagree, 1 = strongly disagree). Items 10, 11 and 12 should be reversely coded [26]. The difference will be measured on the overall scale.

Also, a secondary outcome, Safety and Attitude Questionnaire (SAQ) is a self-reported assessment of attitude towards patient safety with 60 items to be rated on a 5-point Likert scale, 5 = Agree strongly, 4 = Agree slightly, 3 = Neutral, 2 = Disagree slightly, 1 = Disagree strongly). Items two and 11 are negatively worded [23, 27]. We will use the Danish version of SAQ [23].

The final secondary outcome is difference in knowledge evaluated using MCQ; evaluation of participants’ attitudes about the CBL sessions; qualitative content analysis on interactions between healthcare professionals during the CBL sessions.

### Data collection

This study is ongoing with recruitment starting in September 2019; intervention begins on February 25th to April 1st, 2020. Data collection ends approximately August 2020.

Figure 3 and Table 6 lists timepoint of measurement and collection of outcomes.

**Table 6:** Collection of outcomes

Schedule	2–4 months before intervention	2 months before intervention	1–2 months before intervention	Intervention: Case-based learning	1–3 months after intervention
Information for all staff	Oral information at meetings and conferences about case-based learning	Written and oral information by individual email to all staff	Oral information for participants interested in randomisation		
Trial participants			Participants will be included consecutively.		
Trial participants			Pre AITCS Pre RIPLS Pre SAQ	MCQ on gastrointestinal side effects Course evaluation	Post AITCS Post RIPLS Post SAQ
Data collection on participants			Demographic data: sex, age, profession and years of work	Observations of interaction: who speaks, when and about what	

*AITCS* Assessment of Interprofessional Team Collaboration Scale, *MCQ* multiple-choice quiz, *RIPLS* Readiness for Interprofessional Learning Survey, *SAQ* Safety Attitude Questionnaire

### Sample size

Data on the effectiveness of interprofessional education for making the sample size calculation are sparse [21, 28–30]. We have chosen to calculate the required sample size based on previous interprofessional cluster randomised studies [21, 30].

We assume that the primary outcome will be normally distributed with a standard deviation of 20 points [30]. Using a t-test to compare the means in the experimental and the control group and assuming a mean difference of 12 [30], a power of 80% and a significance level of 5%, we need 45 participants in each group.

### Sample size estimation adjusted for clustering

Observations on participants on the same team will be correlated [31], which means the effective sample size will depend on the intraclass correlation coefficient (ICC) [31]. To adjust the sample size, the crude sample size needs to be multiplied by the design effect. The average cluster size is 13, and we assume the ICC to be 0.05.

Design effect =1 + (cluster size – 1) x ICC → design effect =1.6. Accordingly, the sample size will be 45 × 1.6 = 72 participants in each arm. With at least 80 participants in each group, there is room for up to 10% missingness.

### Allocation and sequence generation

Blinded randomisation to ICBL (experimental group) and monoprofessional CBL (control group) will be performed centrally by a computer algorithm. To ensure representation of the smaller professional groups in the experimental groups, they will be randomised separately, before randomising the nurses and doctors. MKTOP and JN will generate the allocation sequence that allocates participants to the sessions by the computer algorithm. MKTOP will subsequently contact and enrols participants, and assign them to the session they were randomly selected to participate in. The healthcare professionals, the educators providing the educational intervention, and the researchers analysing the recordings will not be blinded to the intervention. The allocated intervention group will be blinded for the data managers and statisticians.

### Statistical methods

We only expect a small number of missing observations (due to illness, rescheduling, absences) and will assume that these will be missing at random, [32] in which case the linear mixed model is valid, if correctly specified.

For all outcomes, the group means in the experimental and the control group postintervention will be compared, adjusting for the preintervention scores using a linear mixed model that includes the postintervention as outcomes, assuming the group means at baseline to be equal due to randomisation (a constrained linear mixed model [33]). Analyses will be performed unadjusted and adjusted for sex, age, profession and years of work.

### Data handling and record keeping

Each participant will be registered with an individual trial number known only to the PhD student designing the trial. The study is blinded to all other investigators and management. Participant attendance in the CBL session and completion of their questionnaires will be kept track of. Data on demography and results from questionnaires will be stored in REDCap. The MCQ hardcopies will be archived under the same unique trial number and stored in a locked cabinet at Rigshospitalet.

### Quality control and quality assurance

The trial will be monitored internally only.

## Ethics and dissemination

Participants will be exclusively healthcare professionals or professionals trained to work with children and adolescents with cancer. No patients will be involved in the trial.

### Risk and benefits

There is no anticipation of harm or risk; however, some potential stress, such as fear of exposing one’s own lack of knowledge to colleagues may occur. The departments involved in the trial have a well-established system to provide psychological help to staff involved in emergencies, and this system can be activated if trial participants unexpectedly require psychological support.

### Ethical considerations

The trial will comply with the General Data Protection Regulation. Relevant approval by the Danish Data Protection Agency has been obtained. The trial is exempt from approval by the National Committee on Health Ethics Research (http://en.nvk.dk/how-to-notify/what-to-notify). The trial is registered at ClinicalTrials.gov.

Participants will be assured that their personal data, data on questionnaires and audio recordings will remain anonymous during analysis and reporting. The participants will be asked to respect the confidentiality of their observations about colleagues’ participation in the CBL session.

### Publication plan

The study will adhere to the International Committee of Medical Journal Editor’s guidelines on authorship.

This randomised trial is part of Martha Krogh Topperzer’s PhD project entitled: “Interprofessional education in childhood cancer”. In addition to a publication resulting from this trial, other publications [8, 9] will comprise her PhD dissertation.

### Publications planned

#### Design articles

“Interprofessional versus monoprofessional case-based learning in childhood cancer and the effect on healthcare professionals’ interprofessional attitudes and knowledge: study protocol for a randomised controlled trial”.

“Interprofessional case-based learning improves healthcare professionals’ interprofessional attitudes in childhood cancer: a randomised controlled trial”.

“Intra-professional interactions: findings from interprofessional case-based learning in childhood cancer”.

All results from the trial (negative, positive and inconclusive) will be published in a scientific journal or, alternatively, in a report and online.

Juliane Marie Centre for children, women and reproduction, Rigshospitalet, University of Copenhagen, Denmark is responsible for the intervention.

## Discussion

To our knowledge, this is the first randomised trial investigating the effect of interprofessional versus monoprofessional CBL on healthcare professionals’ interprofessional attitudes.

This study will provide insight into the differences between interprofessional and monoprofessional CBL and how it affects the healthcare professionals’ interprofessional collaboration and attitudes. Moreover, this study will contribute data on teaching methods for interprofessional teamwork skills, as the evidence in this area is sparse [34].

A potential limitation of this trial is that it is a single-centre trial that includes only a moderate number of participants. There is a risk of contamination among participants as some of the healthcare professionals work together and can influence each other across randomised groups. At the CBL session, participants will be told not to speak about the intervention with their colleagues as doing so may interfere with the trial results.

Another limitation is that there are no clinical outcomes such as decreased scores of gastrointestinal side effects because the primary outcome is a test of healthcare professionals’ attitudes. Provision of knowledge does not necessarily result in practice change [35]. Not only does practice change happen slowly, but the healthcare system is inherently complex, with multiple confounding factors such as the rapid turnover of healthcare professionals, maternity leave and postgraduate education for doctors, for example [36].

Interventions directed at the behaviour of healthcare professionals are categorised as complex because they consist of various interconnecting components [37]. The causality of health education interventions is multifactorial, making it difficult to reproduce exactly. Designing a framework for evaluating complex interventions may represent a relevant step in ensuring the continued implementation of an interprofessional curriculum.

## Supplementary Information

**Additional file 1.**

## Data Availability

All data generated and analysed during this study will be available in subsequently published articles. This study has not been submitted to or published at any other journal.

## Abbreviations

AITCS: Assessment of Interprofessional Team Collaboration Scale
CBL: Case-based learning
ICC: Intraclass correlation coefficient
ICBL: Interprofessional case-based learning
MCQ: Multiple-choice quiz
RIPLS: Readiness for Interprofessional Learning Survey
SAQ: Safety Attitude Questionnaire
